# Efficient long-term survival of cell grafts after myocardial infarction with thick viable cardiac tissue entirely from pluripotent stem cells

**DOI:** 10.1038/srep16842

**Published:** 2015-11-20

**Authors:** Takehiko Matsuo, Hidetoshi Masumoto, Shuhei Tajima, Takeshi Ikuno, Shiori Katayama, Kenji Minakata, Tadashi Ikeda, Kohei Yamamizu, Yasuhiko Tabata, Ryuzo Sakata, Jun K. Yamashita

**Affiliations:** 1 Department of Cell Growth and Differentiation, Center for iPS Cell Research and Application (CiRA), Kyoto University, Kyoto, Japan; 2 Laboratory of Stem Cell Differentiation, Stem Cell Research Center, Institute for Frontier Medical Sciences, Kyoto University, Kyoto, Japan; 3 Department of Cardiovascular Surgery, Kyoto University Graduate School of Medicine, Kyoto, Japan; 4 Department of Biomaterials, Institute for Frontier Medical Sciences, Kyoto University, Kyoto, Japan

## Abstract

Poor engraftment of cells after transplantation to the heart is a common and unresolved problem in the cardiac cell therapies. We previously generated cardiovascular cell sheets entirely from pluripotent stem cells with cardiomyocytes, endothelial cells and vascular mural cells. Though sheet transplantation showed a better engraftment and improved cardiac function after myocardial infarction, stacking limitation (up to 3 sheets) by hypoxia hampered larger structure formation and long-term survival of the grafts. Here we report an efficient method to overcome the stacking limitation. Insertion of gelatin hydrogel microspheres (GHMs) between each cardiovascular cell sheet broke the viable limitation via appropriate spacing and fluid impregnation with GHMs. Fifteen sheets with GHMs (15-GHM construct; >1 mm thickness) were stacked within several hours and viable after 1 week *in vitro*. Transplantation of 5-GHM constructs (≈2 × 10^6^ of total cells) to a rat myocardial infarction model showed rapid and sustained functional improvements. The grafts were efficiently engrafted as multiple layered cardiovascular cells accompanied by functional capillary networks. Large engrafted cardiac tissues (0.8 mm thickness with 40 cell layers) successfully survived 3 months after TX. We developed an efficient method to generate thicker viable tissue structures and achieve long-term survival of the cell graft to the heart.

Recent progress in stem cell biology and tissue engineering are contributing to the development of various cell transplantation (TX) strategies. Nevertheless, a high and critical hurdle in tissue engineering is the size of viable cell mass through simple diffusion^1^,^2^,^3^,^4^,^5^,^6^,^7^,^8^. Furthermore, continuous beating of the heart and limitation in cardiomyocyte proliferation make it difficult to generate large and well survived cardiovascular tissue structures in the heart by cell TX strategies. Thus, how to improve engraftment efficiency is a major challenge in cell TX strategies in the cardiac field^9^,^10^,^11^,^12^,^13^.

A cell sheet technology using a temperature-responsive culture surface coated by poly (N-isoproplyacrylamide) (PIPAAm)^14^,^15^ is an innovative method for the generation of 3D tissue. The culture surface is hydrophobic at 37 °C, which makes it amenable for cells to attach on the dish, but becomes hydrophilic at room temperature, which allows cells to detach from the dish as a sheet-like structure. This cell sheet, which holds approximately 3-4 cell layers, can be directly applicable to cell TX^16^,^17^. However, in practice, 3 sheets (approximately 80 μm thickness) is the upper limit for stacking due to hypoxic cell damage^15^,^18^.

We have been investigating cardiovascular cell differentiation and regeneration using pluripotent stem cells^16^,^17^,^19^,^20^,^21^,^22^,^23^,^24^,^25^. We have succeeded in systematically inducing various cardiovascular cells, i.e. cardiomyocytes (CMs), endothelial cells (ECs), and vascular mural cells (MCs), from common progenitor Flk1^+^ cells^19^,^23^,^24^. Recently, we engineered cell sheets with defined cardiovascular cell populations entirely from mouse embryonic stem cells (ESCs) and human induced pluripotent stem cells (iPSCs), and transplanted 3-sheet stacked constructs into a rat myocardial infarction model^16^,^17^. Though cardiac function was ameliorated after the sheet transplantation, the sheet survival after TX was limited to one month in the mouse ESC study^16^. Thus, new technology for the real regeneration of neo-cardiac tissue is needed.

Gelatin hydrogel is a biodegradable biomaterial that possesses excellent capacity as a cell culture substrate. As the water content of gelatin hydrogel is more than 95%^26^, culture media and body fluids containing oxygen and nutrients can be easily impregnated into gelatin hydrogel^27^,^28^,^29^. We recently reported that the addition of gelatin hydrogel microspheres (GHMs) into cell aggregates can improve cell survival in culture^27^. In the present study, we applied GHM technology to stack up cardiovascular cell sheets and succeeded in generating thick viable cardiac structures entirely from pluripotent stem cells. These structures were transplanted and formed organized and long-lasting cardiac tissue *in vivo*.

## Methods

### Reagents

Recombinant human vascular endothelial growth factor 165 amino-acid isoform (VEGF_165_) and 8-bromoadenosine-3′,5′-cyclic monophosphate sodium salt (8bromo-cAMP) were purchased from R & D systems (Minneapolis, MN), and Nacalai Tesque (Kyoto, Japan), respectively. Gelatin for culture dish coating was purchased from Sigma-Aldrich (St. Louis, MO). Mitomycin-C was purchased from Kyowa Hakko Kirin (Tokyo, Japan). Cyclosporin-A was purchased from Wako Pure Industries, Ltd. (Osaka, Japan), and dissolved in dimethyl sulfoxide (DMSO) (Nacalai Tesque, Kyoto, Japan).

### Antibodies

Monoclonal antibodies for murine vascular endothelial (VE)-cadherin (VECD1) and murine Flk1 (AVAS12) were prepared and labeled in our laboratory as described previously^23^,^24^. Phycoerythrin (PE)-conjugated CD31 was purchased from BD Biosciences-Pharmingen (San Diego, CA) and used for flow cytometry analysis. The following antibodies were used as first antibodies for immunostaining: monoclonal antibodies for cardiac troponin-T (cTnT) (1:2000 for immunofluorescence [IF], 1:500 for immunohistochemistry [IHC]; Thermo Scientific, Fremont, CA), CD31 (1:500 for IHC; BD Pharmingen), α-smooth muscle actin (αSMA) (1:200 for IF; Sigma-Aldrich), and polyclonal antibodies for von Willebrand factor (vWF) (1:2000 for IF; DAKO, Carpinteria, CA), and HIF1 alpha (1:200 for IF; GeneTex). The following antibodies conjugated with Alexa florescent dye were purchased from Invitrogen and used as second antibodies for immunostaining: anti-mouse IgG-Alexa546, anti-mouse IgG-Alexa594, anti-rat IgG-Alexa488, anti-rat IgG-Alexa546, and anti-rabbit IgG-Alexa488 (1:500 for IF). For cTnT-staining in cardiovascular cell sheets, sections were incubated for 60 minutes with primary antibody at room temperature, and then applied to EnVision^TM^+ System-HRP (DAB) (DAKO) according to the manufacturer’s instructions. For CD31 staining, sections were applied to Histofine Simple Stain (Nichirei Biosciences Inc., Tokyo, Japan).

### Mouse ESC culture

Two mouse ESC sublines from E14tg2a cell line were used as previously described^16^. Briefly, EMG7 mouse ESC line that carries the mouse α-myosin heavy chain (MHC) promoter-driven eGFP gene was used for the differentiation of CMs^24^,^30^. EStTA-ROSA mouse ESC line was used for the differentiation of ECs and MCs^20^.

### Mouse ESC differentiation

Induction of and sorting for Flk1^+^ cells were performed based on our previous reports^16^,^23^,^24^. In summary, mouse ESCs were cultured in differentiation medium (DM) (alpha minimum essential medium [αMEM] [GIBCO, Grand Island, NY] supplemented with 10% fetal bovine serum [FBS] and 5.5 mmol/L 2-mercaptoethanol) without leukemia inhibitory factor (LIF) on gelatin-coated dishes for 96–112 hours. For the differentiation of CMs, purified Flk1^+^ cells were plated onto mitomycin-C-treated confluent OP9 cell sheets (MMC-OP9) and cultured in DM to induce CM differentiation. Cyclosporin-A (3 μg/mL) was added to Flk1^+^ cell culture to promote CM differentiation^30^. For the differentiation of ECs or MCs, purified Flk1^+^ cells were plated onto gelatin-coated dishes, and then cultured with DM in the presence of VEGF_165_ (50 ng/mL) and 8bromo-cAMP (0.5 mmol/L)^31^.

### Flow cytometry analysis and cell sorting

After 4 days culture of Flk1^+^ cells on MMC-OP9, cultured cells were harvested and subjected to cell sorting with FACS (fluorescence-activated cell sorting) (Aria III, BD Biosciences, Franklin Lakes, NJ). Viable GFP-positive cell population was evaluated and sorted as differentiated CMs (GFP^+^ CMs)^19^,^24^,^30^. ESC-derived ECs and MCs were selectively induced and harvested on the third day of Flk1^+^ cell culture on gelatin-coated dishes with VEGF and 8brom-cAMP (Flk-d3 EC/MC)^21^,^31^.

### Mouse ESC-derived cardiovascular cell sheet generation

Cell sheets with defined cardiovascular cell populations were generated as previously described^16^. In brief, Flk1^+^ cells induced from EStTA-ROSA cells were plated onto gelatin-coated 12- or 24-multiwell temperature-responsive culture dishes (UpCell) (CellSeed, Inc., Tokyo, Japan) at 37 °C in a humidified atmosphere containing 5% CO_2_. The cells were plated at 2.5–4.0 × 10^4^ cells/well in a 12-multiwell UpCell or 1.4-2.2 × 10^4^ cells/well in a 24-multiwell UpCell with 1 mL of DM containing VEGF_165_ (50 ng/mL) and 8bromo-cAMP (0.5 mmol/L) to selectively induce ECs and MCs. After 3 days of EC and MC induction, separately prepared Flk-d3 EC/MC (5.0 × 10^5^ cells for 12 well, 2.2 × 10^5^ cells for 24 well dish) and GFP^+^ CMs (5.0 × 10^5^ cells for 12 well, 2.2 × 10^5^ cells for 24 well dish) were plated onto the UpCell dishes (i.e. purified C + E + M cells onto E + M underlying cells; C: CMs, E: ECs, M: MCs). After 4 days, the cultured cells were transferred to room temperature for 15–30 minutes to release the cultured cells as intact monolayer sheets.

### Preparation of gelatin hydrogel microspheres

GHMs were prepared by dehydrothermal cross-linking of gelatin microspheres prepared in a water in oil emulsion state according to a method previously reported^26^,^32^. Briefly, an aqueous solution (20 mL) of 10 wt% gelatin (isoelectric point 5.0, weight-averaged molecular weight 100,000, Nitta Gelatin Inc., Osaka, Japan) was preheated at 40 °C, followed by stirring at 200 or 400 r.p.m. for 10 min to prepare water in oil emulsion. The emulsion temperature was decreased to 4 °C for natural gelation of the gelatin solution to obtain non-crosslinked microspheres. The resulting microspheres were washed three times with cold acetone in combination with centrifugation (5000 r.p.m., 4 °C, 5 min) to completely exclude residual oil. Then they were fractionated by size using sieves with apertures of 20, 32, and 53 μm (Iida Seisakusho Ltd, Osaka, Japan) and air dried at 4 °C. The non-crosslinked and dried gelatin microspheres (200 mg) were treated in a vacuum oven at 140 °C and 0.1 Torr for dehydrothermal crosslinking of gelatin according to a previously reported method^26^,^32^.

### Stacking cell sheets

**Figure 1:**
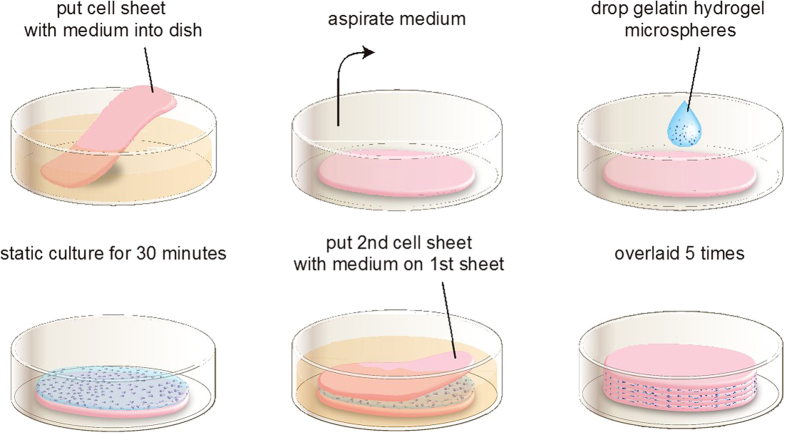
Cardiovascular cell sheet stacking with GHMs *in vitro*. Process of cardiovascular cell sheet stacking. The first cardiovascular cell sheet is put into a dish with medium. The sheet is spread out on the dish during medium aspiration. GHM-containing solution is added drop-wise and spread on the surface, and then incubated at 37 °C in the absence of medium for 30 minutes. A second cardiovascular cell sheet is then applied on the first sheet with medium. The same procedure is repeated until 5 layers are stacked.

Spontaneously detached cell sheets were suspended in aqueous media. To stack the cell sheets, one cell sheet with culture media was gently aspirated into the tip of a 5 or 10 mL-pipette and transferred onto a new gelatin-coated 60 mm or 35 mm culture dish. After spreading the cell sheet without folds by aspirating the medium, PBS with GHMs (100 μg/μL) or PBS alone was put and spread on the surface of the cell sheet (5 μL and 2.5 μL for 12-multiwell and 24-multiwell UpCell-derived sheets, respectively). The dishes were incubated at 37 °C for 30 min to allow the cell sheet to adhere to the culture surface. Then a second cell sheet was placed on top of the first and attached without folds by aspirating the media. In a similar way, quintuple-stacked constructs (5-constructs) were generated on the culture dishes (Fig. 1). Before TX, the constructs were pre-stained with Hoechst 33342 (Life Technologies Carlsbad, CA) for 30 minutes and washed with DM. The constructs were detached from the culture dish by flushing several times with culture medium using a 1000 μL micropipette. To obtain 10-constructs, a second 5-construct was overlaid on the first one, and to obtain 15-constructs, a third 5-construct was overlaid on the 10-construct.

### Live/Dead assay

A layered cardiovascular cell sheet was used to evaluate cell viability. Seven days after cultivation, the constructs were incubated with staining solution (50 mL/L Ethidium Homodimer III and 50 mL/L Hoechst 33342, PromoCell GmbH, Heidelberg, Germany) in a pH-adjusted buffer for 15 minutes at room temperature and protected from light. Fluorescent images were obtained using a microscope (Biorevo BZ-9000, Keyence, Osaka, Japan).

### TUNEL assay

Seven days after stacking cardiovascular cell sheets, apoptosis was assessed in cross sections with TUNEL assay (Click-iT Plus TUNEL Assay, Invitrogen) following the manufacturer’s instruction. Briefly, the cardiovascular sheets were fixed in 4% paraformaldehyde and routinely processed into 5-μm-thick paraffin-embedded sections. These samples were deparaffinezed and fixed in 4% paraformaldehyde again. Then EdUTP nucleotide was incorporated into dsDNA strand breaks by TdT enzyme and attached an Alexa Fluor picolyl azide dye. Fluorescent images were obtained using a microscope (Biorevo BZ-9000, Keyence). The numbers of TUNEL-positive cells and DAPI was calculated by Image-J software.

### Animals

All animals including NOD-SCID mice (10 weeks old, male) and F344/NJcl-*rnu/rnu* rats (athymic nude rats; 8-10 weeks old, male) were purchased from CLEA Japan (Osaka, Japan). All animal experimental protocols were approved by the Animal Experimentation Committee, Kyoto University. All animal experiments were performed according to the *‘Guidelines for Animal Experiments of Kyoto University’*, which confirms to Japanese law and *‘the Guide for the Care and Use of Laboratory Animals’*.

### Myocardial infarction

Male athymic nude rats aged between 10–12 weeks were housed in a controlled environment. Myocardial infarction (MI) was induced in isoflurane-anaesthetized rats by permanent ligation of the left anterior descending coronary artery as previously described^16^,^33^,^34^. Rats of which hearts showed more than 30% left ventricular (LV) fractional shortening (FS) in echocardiograms 6 days after ligation were excluded from subsequent experiments. Hearts were harvested at 1 to 12 weeks after treatment/sham operation and prepared for immunofluorescence, immunohistochemistry and species-specific fluorescent *in-situ* hybridization (SS-FISH).

### GHM-constructs transplantation

Seven days after inducing MI, each rat was randomly assigned to one of the three groups: GHM-construct TX group, control-construct TX group, and sham group. In the former two groups, five-cardiovascular cell sheet constructs with or without GHMs were applied to the surface of the anterior wall of the heart as previously described^16^. In summary, the constructs were spread manually to cover the whole MI area and the border area and stably placed onto the surface of the heart without sutures. The chest was closed 15–20 minutes after surgery. In sham-operated group, the chest was closed 15–20 minutes after thoracotomy.

### Cardiac function assessment

To assess global cardiac function and left ventricle (LV) size, echocardiograms were performed with the Vivid 7 system (GE Healthcare, Waukesha, WI) and an 11-MHz imaging transducer (GE 10S ultrasound probe, GE Healthcare). Echocardiograms were performed before ligation (baseline), and on day 6 (pre TX, i.e., 6 days post-MI), and 1, 2, 4, 8, and 12 weeks after TX by an independent person in a blinded fashion as previously described^16^,^33^,^34^. Diastolic and systolic area of LV (LVAd, LVAs), diastolic lengths of LV inner circumference (CIRCd) and those of akinetic area in diastole (SCAR) were recorded and measured with B-mode examination. Values were calculated as follows:

Fractional shortening (FS) (%) = (LVDd–LVDs)/LVDd ×100.

Akinetic length (AL) (%) = SCAR/CIRCd ×100.

Besides the experimental model (GHM-construct TX group, control TX group, or sham group), echocardiograms were performed on normal rats, which had no surgical intervention in order to quantify the normal values of the parameters of the lineage/age/weight-matched rats (n = 5).

### Species-specific FISH analysis

FISH probes which recognize and hybridize with sequence repeats specific for each animal species were arranged by Chromosome Science Labo (Sapporo, Japan)^16^,^35^,^36^. The nucleotide probes were applied to the fixed and pre-treated sections that were denatured and then hybridized. Additional IF staining for cTnT and vWF was performed on the FISH samples. Samples were examined by fluorescence microscopy (LSM 710 Laser Scanning Microscopes, Carl Zeiss, Oberkochen, Germany) and Carl Zeiss software.

### Histological analyses

For cross-sectional observation, cardiovascular cell sheets were fixed in 4% paraformaldehyde and routinely processed into 5-μm-thick paraffin-embedded sections. Hematoxylin and eosin (HE) staining was performed using conventional methods as previously described^16^,^33^,^34^. For cTnT-staining, sections were incubated for 60 min with primary antibody at room temperature, and then applied to LSAB2 kit/horseradish peroxidase (HRP) (diaminobenzidine; DAB) (DAKO) according to the manufacturer’s instructions. Hearts were immersed and perfusion fixed with 4% PFA and embedded in OCT compound (Sakura Finetek Japan, Tokyo, Japan) and frozen. Several 5-micrometer sections were made at 50-μm intervals along the short axis and examined. For IF staining, sections were treated with Protein Block Serum Free (DAKO) and incubated for 60 min with primary antibodies at room temperature. The area of engraftment was calculated as double positive cells for cTnT staining and mouse signal with SS-FISH or as positive cells for Hoechst 33342. For lectin perfusion analysis, rats were received intravenous injections of 1.5 ml of 1 mg/ml DyLight 594-conjugated tomato (Lycopersicon esculentum) lectin (Dye-lectin) (Vector Labs, Burlingame, CA) in PBS into the inferior vena cava 15 min prior to sacrifice. After excision, the hearts were sectioned manually into 5-micrometer that were made at 50-micrometer intervals along the short axis and examined. All immunostained sections were photographed and calculated with Biorevo BZ-9000 or LSM 710 Laser Scanning Microscopes (Carl Zeiss, Oberkochen, Germany).

### Extracellular field potential measurement

Extracellular field potential (EFP) of cell sheet constructs before and after TX was measured by the difference between electric potentials of two sensor electrodes with 1 mm distance (Research electrode SCR-2; unique medical co). The electric potential was amplified (bio-signal amplifier unit; unique medical co) and recorded (UAS-3088; unique medical co). Just before TX of 5-GHM constructs, EFP was measured by direct attachment of the sensor electrodes to the cell sheet constructs in a medium (sheet EFP (*in vitro*)). Then, the constructs were transplanted to the normal hearts of male athymic nude rats (F344/N Jcl-rnu/rnu, CLEA Japan, Inc., Tokyo, Japan) aged 12 weeks. EFP of the transplanted cell constructs was measured by direct attachment of the electrodes on the surface of the cell constructs (sheet EFP (heart)) with simultaneous recording of the rat electrocardiogram (ECG).

### Statistical analysis

Results are presented as mean ± s.e.m. *P* values were obtained with non-parametric statistics; Mann-Whitney U test for a non-parametric test using GraphPad Prism 5 for Windows (version 5.03, GraphPad Software, Inc., San Diego, CL). Comparisons between >2 groups were performed using a Kruskal-Wallis one-way analysis of variance by ranks for a non-parametric test, then by post-hoc comparisons between groups by Steel-Dwass test. Differences were considered statistically significant when *P* < 0.05.

All experimental protocols including animal and recombinant DNA experiments were approved by the Animal Experimentation Committee and Agency of Health, Safety, and Environment, Kyoto University.

## Results

### Stacking of pluripotent stem cell-derived cardiovascular cell sheets with GHM insertion

**Figure 2:**
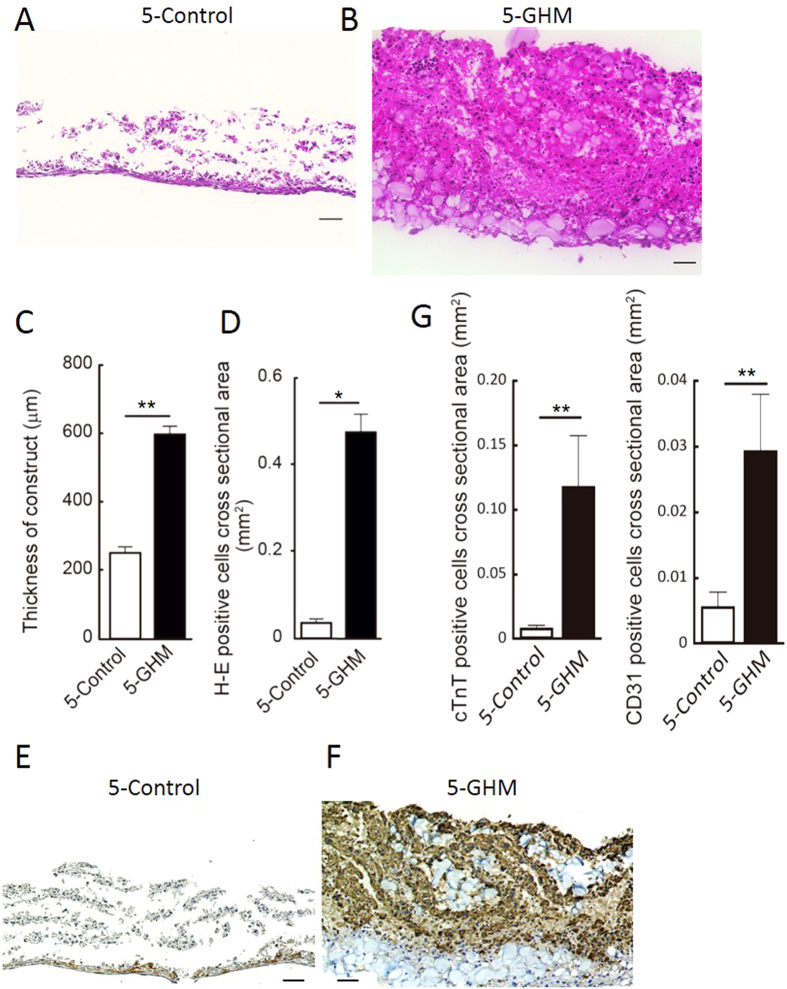
Characteristics of cardiovascular cell sheet constructs. (**A,B**) Cross-sections of stacked sheets with or without 0.5 mg/cm^2^ of GHMs (φ 20–32 μm) inserted between. HE staining after 7-day culture. Scale bars, 50 μm. Magnifications, x200. (**A**) 5-Control: 5-sheet constructs without GHMs. (**B**) 5-GHM: 5-sheets with GHMs. (**C,D**) Thickness of constructs and viable cell area (HE-positive area) after 7-day culture. Mean ± s.e.m. **P < 0.01, *P < 0.05 (Mann-Whitney U-test, n = 5). (**E,F**) Immunostaining for cTnT (brown) after 7-day culture. 5-control (**E**) and 5-GHM (**F**) constructs. Scale bars, 50 μm. Magnifications, x200. (**G**) Quantitative evaluation of cTnT-positive and CD31-positive area. Mean ± s.e.m. **P < 0.01 (Mann-Whitney U test, cTnT, n = 9; CD31, n = 6).

We prepared pure cardiovascular cell populations, i.e. CMs, ECs and MCs, from Flk1^+^ cardiovascular progenitor cells in our mouse ESC differentiation system, and generated cardiovascular cell sheets by re-assembling them as previously reported^16^. These sheets consisted of CMs (45.6 ± 3.8% of total cells), ECs (9.9 ± 1.7%), and MCs (41.8 ± 3.3%) (n = 14, FACS-analyzed) (Supplementary Fig. S1). Total cell count of each sheet (generated in 12-well UpCell dishes) was 3.73 ± 2.7 × 10^5^ on average (n = 14). We then manually stacked the cell sheets one by one with the insertion of GHMs by adding drop-wise and spreading the GHM solution onto the entire surface of the sheet (Fig. 1). We optimized doses and sphere sizes of GHMs (Supplementary Fig. S2). Insertion of approximately 0.5 mg/cm^2^ of GHMs (φ 20–32 μm) achieved highly viable stacked cell sheets even after 1 week of culture (Fig. 2A,B). Cell constructs of 5-sheets with GHMs (5-GHM constructs) showed dramatically thicker wall (597.1 ± 23.95 vs. 250.9  ±  17.7 μm, n = 5, **P < 0.01, *P < 0.05; Fig. 2C) and greater viable cell area (0.4648  ±  0.03102 vs. 0.03511  ±  0.009244 mm^2^, n = 5, *P < 0.05; Fig. 2D) than 5-sheet constructs simply stacked without GHMs (5-control constructs). Immunostaining for cTnT and CD31 demonstrated that CM and EC contents were increased approximately 13.6-times and 5.2-times with GHM insertion than control, respectively (Fig. 2E–G). Thus, GHM insertion efficiently helps to stack cardiovascular cell sheets.

### Mechanisms and roles of GHM insertion

**Figure 3:**
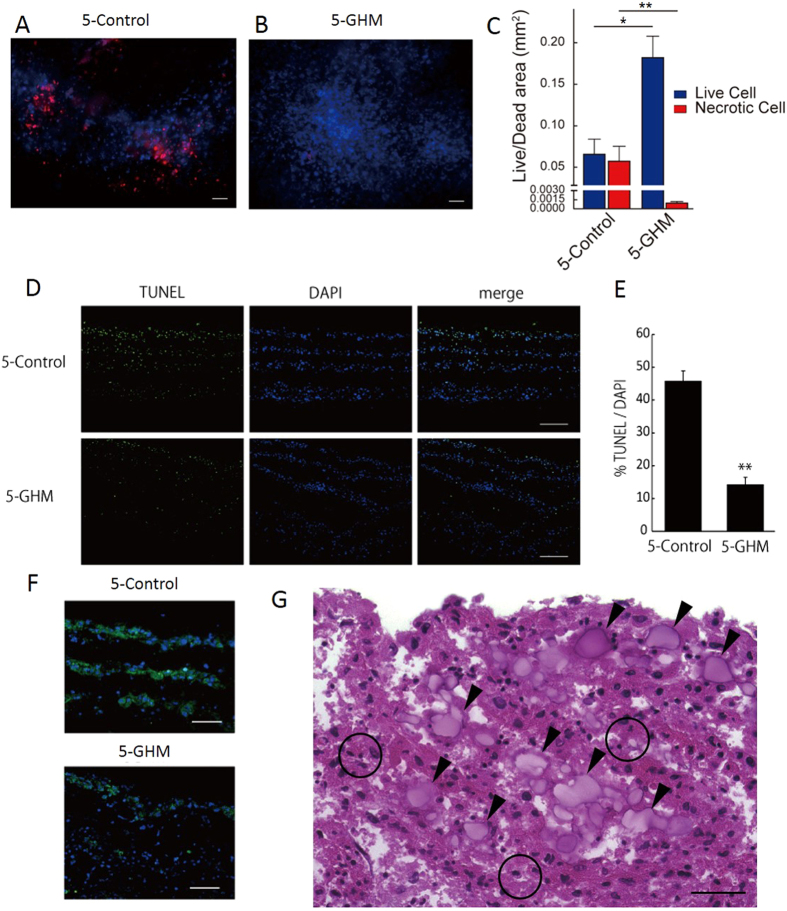
Mechanisms and roles of GHM insertion. (**A,B**) Live/Dead assay after 7-day culture. Live cells (blue), necrotic cells (red). (**C**) Areas in cross sections. **P < 0.01, *P < 0.05 (Mann-Whitney U test, n = 4). Scale bars, 50 μm. (**D,E**) TUNEL assay. (**D**) TUNEL staining in cross sections after 7-day culture. TUNEL (green), DAPI (blue). Scale bars, 100 μm. (**E**) Percentage of TUNEL-positive cells in total cells (DAPI). **P < 0.01 (Mann-Whitney U test, 5-control, n = 4; 5-GHM, n = 3). (**F**) HIF1α immunostaining after 7-day culture. HIF1α (green), DAPI (blue). Scale bars, 50 μm. (**G**) A higher magnification view of Fig. 2B. Arrowheads indicate GHMs. Open circles indicate direct contacts between one cardiovascular sheet and the other. Scale bar, 30 μm. All photos are taken with x200 magnification.

We next examined the mechanisms and roles of GHMs in the efficient cell sheet stacking. First we evaluated cell survival with Live/Dead assay. Whereas half of the cells became necrotic after 1-week culture of control constructs, almost all cells were alive in 5-GHM constructs (Fig. 3A–C). TUNEL assay also demonstrated efficient reduction of apoptosis in 5-GHM constructs *in vitro* (Fig. 3D–E). Whereas approximately 45% of nuclei were positive for TUNEL after 1-week culture in control constructs, GHM insertion significantly reduced TUNEL-positive nuclei to approximately 15% of total nuclei, indicating that GHMs dramatically reduced apoptosis after cell sheet stacking. We further examined hypoxic status in the cultured cell sheets with hypoxia-inducible factor 1α (HIF1α) expression (Fig. 3F). Although HIF1α was broadly expressed in stacked cell layers in the control after 1-week culture, the expression was largely reduced in 5-GHM constructs, suggesting that hypoxic status in the stacked cell sheets can be reduced with GHM insertion. Higher magnification view of 5-GHM constructs (Fig. 3G) showed scattered insertion of GHMs between each cardiovascular cell sheet (arrowheads in Fig. 3G). GHMs should provide appropriate spaces between cell sheets and be impregnated with culture media, which should allow oxygen and nutrients to reach each cell sheet broadly. In addition, GHMs did not block the direct contact between each sheet (circles in Fig. 3G), which resulted in synchronous beating of whole cell constructs. We confirmed electrical coupling between the stacked cell sheets by directly measuring EFP of the stacked cell sheet constructs (sheet-EFP). 5-GHM constructs in a culture dish showed spontaneous contraction with a single synchronous sheet-EFP wave (Supplementary Fig. S3a, d). Thus, all these data suggest that GHM insertion can endow ideal features as cardiac tissue, thick viable structure and electrical coupling, on the stacked cardiovascular cell sheet constructs.

### Efficient generation of thick viable cardiac cell structures with GHM method

**Figure 4:**
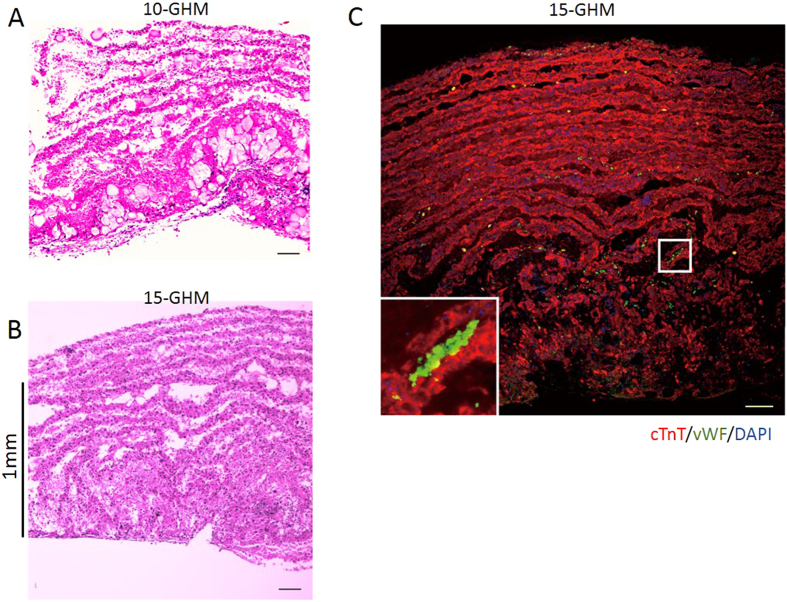
Efficient generation of thick viable cardiac cell structures with GHM method. Thick GHM constructs with 10–15 cardiovascular cell sheets *in vitro* after 7-day culture. (**A,B**) HE staining. Note that construct thickness reaches more than 1 mm in 15-GHM. (**C**) cTnT (CMs; red), vWF (ECs; green) and DAPI (blue) staining. cTnT-negative/vWF-negative/DAPI-positive cells can be estimated as MCs. Inset: clear viable CM layers (red) include ECs (green). Scale bars, 100** **μm. All photos are taken with x100 magnification.

This simple and easy procedure of the GHM method is amenable to generate much thicker sheet constructs. We prepared a number of 5-GHM constructs and then stacked them to generate 10- or 15-GHM constructs within several hours. After 1-week culture of 10- or 15-GHM constructs, viable thicker cardiovascular cell sheet constructs were successfully generated (Fig. 4). The thickness reached more than 1 mm on average in 15-GHM constructs (10-GHM, 911 ± 19.8 μm; 15-GHM, 1050 ± 16.2 μm; n = 4). The thickness of cellular components (HE-positive area/sheet length) in 10-GHM and 15-GHM constructs was calculated to 529 ± 55.5 μm and 670 ± 22.3 μm on average (n = 4), respectively (Fig. 4A,B). Immunostaining for cTnT (CMs) and vWF (ECs) clearly demonstrated that the constructs consisted of multi-layered, viable CMs with vascular cells (Fig. 4C). Thus, all-pluripotent stem cell-derived, thick viable cardiovascular cell constructs consisted of multi-layered CMs and vascular cells can be successfully generated *in vitro* by the cell sheet stacking with GHM insertion method.

### Functional improvements of infarcted hearts after GHM-construct TX

**Figure 5:**
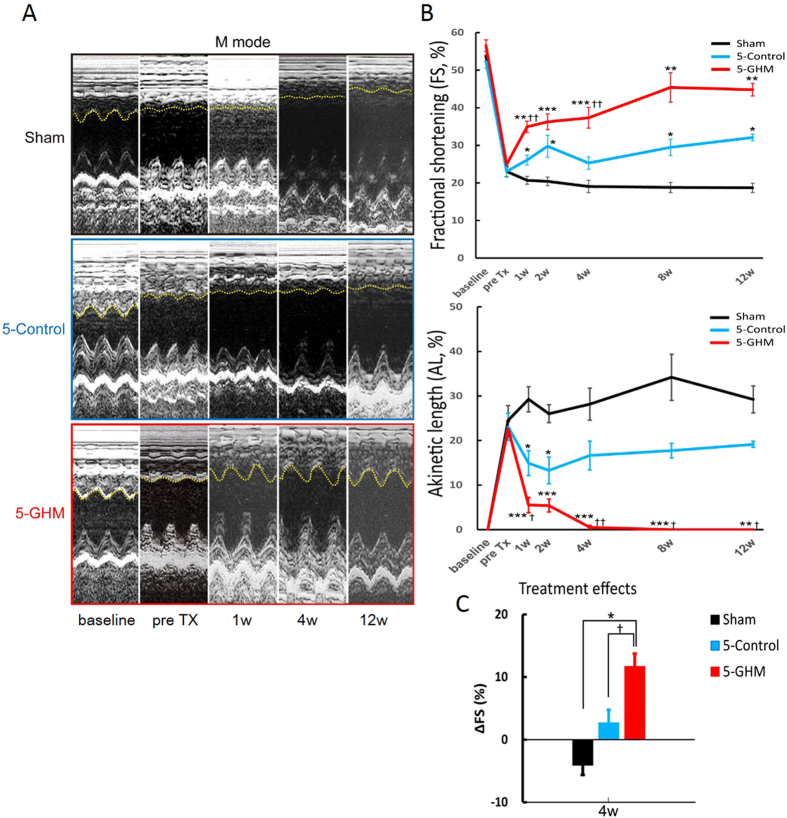
TX of cardiovascular cell sheet constructs to rat infarcted hearts. (**A**) Representative M-mode echocardiographic images at baseline (before MI), pre TX, and 1, 4, and 12 weeks after TX. Yellow dashed lines indicate anterior wall movements. (**B**) FS and AL measured by echocardiogram. Mean ± s.e.m. ***P < 0.001, **P < 0.01, *P < 0.05 versus sham-treated group, ^††^*P* < 0.01, ^†^*P* < 0.05 versus 5-Control group (Kruskal-Wallis test, post-hoc Steel-Dwass multiple comparison test, sham, n = 6; 5-control, n = 11; 5-GHM, n = 10). (**C**) Changes in FS (ΔFS) between pre-TX and 4 weeks. *P < 0.05 versus sham-treated group, †*P* < 0.05 versus 5-Control group (Kruskal-Wallis test, post-hoc Steel-Dwass multiple comparison test, sham, n = 6; 5-control, n = 11; 5-GHM, n = 10).

Next we transplanted the constructs *in vivo*. First we confirmed the survival of the constructs by TX to the subcutaneous spaces of NOD/SCID mice. The constructs were pre-stained with Hoechst 33342 just before TX. Seven days after TX, whereas almost no transplanted cells survived in 5-control construct TX, distinct tissue structures were still preserved in 5-GHM construct TX under the skin (Supplementary Fig. S4). Next we transplanted the constructs (generated in 12-well UpCell dishes) to a myocardial infarction (MI) model. Seven days after the ligation of the left coronary artery (sub-acute MI model), Hoechst-pre-stained constructs were transplanted to cover the infarcted area (Supplementary Fig. S5). We evaluated effects of GHM construct TX on the infarcted hearts by assessing global cardiac function and left ventricle (LV) size with echocardiogram (Table 1). Whereas in the sham-operation group, infarcted wall motion was reduced and cardiac function (FS; fractional shortening) did not change and showed a tendency to further deteriorate until 12 weeks after treatment, 5-control construct TX showed weak beneficial effects on cardiac function (FS) and akinetic length (AL) (Fig. 5A,B, Table 1). 5-GHM construct TX exerted much rapider and greater effects, including significant recovery of FS from 1 week after TX and further improvement towards 12 weeks after treatment. AL also significantly decreased to almost undetectable levels within 4 weeks. 5-GHM construct TX significantly improved FS than sham and control groups at 4 weeks after TX (Fig. 5C, Table 1). Enlargement of systolic diameter of left ventricle (LVDs) was significantly reduced with 5-GHM construct TX than sham group. Diastolic diameter (LVDd) dilatation showed a tendency to be reduced in 5-GHM group (Table 1). Thus, the GHM insertion brought beneficial outcomes in the rapid and long-term functional rescue of infarcted hearts after sheet TX.

### Survival, maturation, and re-organization of GHM-constructs in infarcted hearts

**Figure 6:**
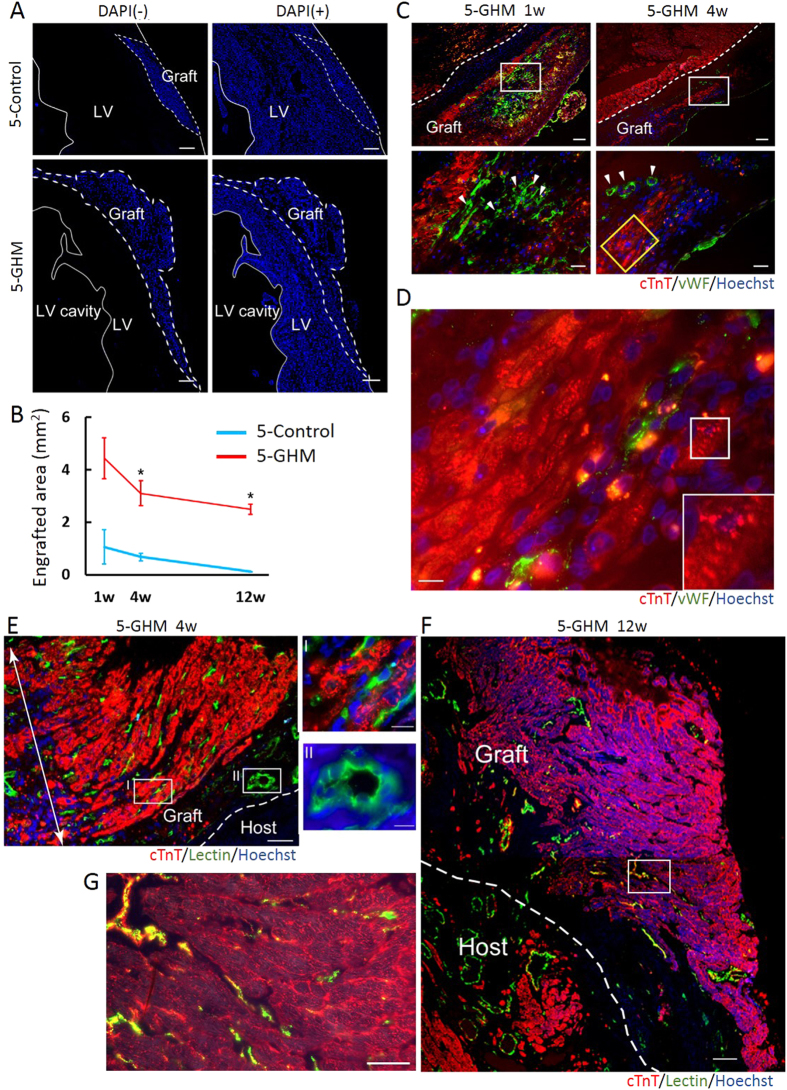
Engraftment status of sheet constructs. (**A**) Nuclei staining images (blue) for pre-stained Hoechst (grafts, left) and DAPI (total cells, right). White dashed lines indicate graft areas. Magnifications, x40. (**B**) Quantitative evaluation of engrafted areas. Mean ± s.e.m. **P < 0.01, *P < 0.05 (Mann-Whitney U test, 1 week: 5-Control, n = 4; 5-GHM, n = 7; 4 weeks: 5-Control, n = 4; 5-GHM, n = 5; 12 weeks: 5-Control; n = 4, 5-GHM; n = 4). (**C**) Immunofluorescence staining for cTnT (red) and vWF (green) of heart sections 1 (left) and 4 (right) weeks after TX of 5-GHMs. Lower panels: high-magnification images (x400) of white boxes in upper panels (x100). White arrowheads: blood vessel formation with vWF-positive cells. Clear vascular-like structures were formed at 4 weeks. (**D**) High-magnification image (x1000) of yellow box in (**C**). Inset: immature sarcomere formation in white box in (**D**). (**E,F**) Connection of grafts with systemic circulation. CMs (cTnT, red), perfused vessels (lectin-stained, green), pre-stained graft nuclei (Hoechst, blue). (**E**) Four weeks after TX. Note that thick neo-CM layers (>20 layers; white double-headed arrow) supported by dense perfused capillary networks (green) were formed. Magnification, x200. Capillaries (I) and larger vessels (II) in the graft (green) were largely Hoechst-positive. Magnification, x1000. (**F**) Twelve weeks after TX. A Large graft (>0.8 mm thickness, >40 cardiac cell layers, in maximum) survived. Magnification, x200. (**G**) High-magnification image (x400) of white box in (**F**). A compact CM tissue with capillary vessels was formed. Scale bars, 300 μm (**A**), 100 μm (C, upper and F), 50 μm (**E,G**), 30 μm (C, lower), and 10 μm (D, E-I and II).

Next, we examined the engraftment status of the sheet constructs. Our previous results^16^ as well as others^37^ indicated that pluripotent stem cell-derived sheets almost disappear within one or two months after TX to infarcted hearts. In this study, a larger engraftment (Hoechst-positive cells) with 5-GHM constructs was observed from 1 week after TX (Fig. 6A) and was sustained until 12 weeks after TX. The estimated engrafted areas at 1 week or 4 weeks after TX were approximately 5 times more than those of 5-control constructs (1 week, 4.44 ± 0.78, n = 7 vs. 1.06 ± 0.65 mm^2^, n = 4, *P* < 0.05; 4 weeks, 3.10 ± 0.47, n = 5 vs. 0.67 ± 0.15 mm^2^, n = 4, P < 0.01, Fig. 6B). Whereas survived area was reduced to an undetectable level in the 5-control construct group until 12 weeks after the TX, a substantial amount of graft was maintained in the 5-GHM construct group (12 weeks, 2.48 ± 0.19 vs. 0.11 ± 0.0054 mm^2^, n = 4, P < 0.01, Fig. 6B). Decrease rate in survived graft area was decelerated after 4 weeks to 12 weeks of TX, suggesting further long-term survival of the graft could be expected. Staining for cardiovascular cell types demonstrated tissue remodeling occurred after the construct TX. Neovascularization mainly with host ECs was induced in close proximity to the grafts compatible with our previous 3-control sheet TX^16^ (Supplementary Fig. S6). In addition, blood vessel formation was also observed in the survived grafts. ECs had accumulated at 1 week and formed clear lumen structures in the grafts at 4 weeks after TX, respectively (Fig. 6C). The majority of non-CM/non-EC cells in the grafts were αSMA-positive mural cell population (Supplementary Fig. S7). Many CMs survived and formed layers of neo-CMs that started to show sarcomere formation though it was still immature (4 weeks, Fig. 6D). We further confirmed connection with the systemic circulation by venous infusion of Dye-lectin that marked perfused vessels (Fig. 6E–G). Surprisingly, drastic re-organization of the grafts was observed at 4 weeks after TX. Various sizes of functional vessels, such as blood vessels with clear lumen and smaller capillaries, were formed in the grafts. The majority of these functional vessels were generated from Hoechst-positive graft-derived ECs (Fig. 6E, I and II). Massive neo-CMs survived and formed large layered structures (>20 layers), which were completely supported by the dense capillary network. Even in 12 weeks after TX, the vascular-supported grafts efficiently survived and formed a large cardiac structure (>0.8 mm thickness, 40 cell layers, in maximum) (Fig. 6F). GHMs that are used in this study was estimated to be degradated *in vivo* around 3 weeks in previous studies^38^. No GHMs were detectable in 12 weeks after TX and instead the grafts were occupied with cellular or tissue components. Compact CM tissue with functional capillary vessels was successfully formed (Fig. 6G). These results indicate that transplanted grafts from pluripotent stem cells were successfully integrated into the heart with blood supply and re-organized into solid neo-CM tissues during the long-term survival.

We finally examined electrical coupling between the graft and host heart after TX. We recorded sheet-EFP and rat electrocardiogram (ECG) simultaneously (Supplementary Fig. S3). 5-GHM constructs before TX showed spontaneous beating with a distinct rhythm from the rat heart beat recorded by ECG (Supplementary Fig. S3a, d). After TX to normal rat heart (Supplementary Fig. S3b), sheet-EFP showed only synchronized rhythm with the rat ECG (Supplementary Fig. S3e), suggesting that the electrical coupling between the graft and host should be established. No increase in sudden death was observed after TX (data not shown), suggesting that lethal arrhythmia should not be induced by TX. Though it is difficult to directly assess actual mechanical support that the graft generated, transplanted GHM constructs may contribute to functional improvement after MI through both paracrine effects and direct contraction.

## Discussion

Combining novel technologies in stem cell biology and tissue engineering, we succeeded in generating vascularized and organized large cardiac tissue from pluripotent stem cells that efficiently survived long-term in infarcted hearts.

Because CMs principally do not proliferate and the heart is continuously beating, cell TX and efficient graft survival in the heart are much more difficult than other cell types or tissues^9^,^10^,^39^. Recently, a mixture of human iPS cell-derived hepatocytes with human umbilical vein ECs and mesenchymal cells was reported to generate 3D tissue structure *in vitro* and *in vivo*^40^. In this case, the cell aggregates could survive and grow on a cranial mesentery, a stable environment. As for cardiac cells, TX of cardiovascular cell sheets to subcutaneous tissue, also a stable environment, showed a long-term survival^41^. In another recent article, needle-injected human ESC-derived CMs were reported to successfully survive and be engrafted in monkey hearts. The thickness of the grafted CMs reached approximately 2 mm in a cross section sample^42^, which is more than two times thicker than our maximum graft thickness after 5-GHM construct TX (approx. 0.8 mm). However, the total transplanted cell number is more than 500 times less in our model than that report (approx. 1.9 × 10^6^ cardiovascular cells vs 10^9^ human CMs). Though it is difficult to directly compare the efficiencies of the engraftments between the two studies because the cell species and TX methods are different, our method has the potential to generate vascularized and long-term surviving thick cardiac tissue in the hearts from a relatively small numbers of transplanted cells.

Additionally, significant and sustained cardiac functional recovery after TX was observed in our study, suggesting a potential for the application of the strategy to cardiac regenerative therapy. Of course, whether our strategy can be extended to larger animals using human pluripotent stem cells requires further study. Based on our human induced pluripotent stem cell (iPSC) differentiation method to CMs^25^,^43^, we recently developed a new and efficient cardiovascular cell differentiation method from human iPSCs, in which cardiovascular populations (CMs, ECs, and MCs) are simultaneously induced as a cell mixture. We generated 3-stacked human iPSC-derived cardiovascular cell sheets and confirmed functional improvements after TX to a rat myocardial infarction model^17^. In that study, we observed some cases in which relatively larger grafts survived 1-2 months after TX, suggesting that human iPSC-derived cardiovascular cell sheets may possess higher survival features than mouse ESC-derived sheets. We have applied GHMs to human iPSC-derived sheets and already succeeded in generating 5-stacked thicker cardiac cell structures (Supplementary Fig. S8). Clinical relevance of GHM-sheet stacking strategy should be examined with human iPSC-derived cardiovascular cell sheets using larger animal models. Usefulness of the strategy including mechanisms of functional improvement, i.e. paracrine effects and/or mechanical supports, maturation of CMs in the grafts, and safety issues such as arrhythmogenicity and tumorigenicity should be further addressed before moving to the clinical stage.

In our method, cellular components, CMs, ECs, and MCs, are entirely derived from pluripotent stem cells. Recently, generation of 3D cardiac structures with blood vessels was reported using rat primary CM and EC culture cells^18^,^44^,^45^. In those reports, cardiac cell sheets with primary cultured cells were layered on bioreactors that can perfuse the cell sheet culture. However, 3-layer cell sheets were stacked sequentially at 1-week intervals, thus requiring around 20 days to generate a 12-sheet structure with approximately 100 μm thickness *in vitro* and 1 mm *in vivo*^18^,^44^,^45^. Our easy and efficient method can generates large viable 3D cardiac structures all from pluripotent stem cells within several hours in a manner suitable for the industrialization of stem cell medicine assuming large-scale and stable cell preparation.

Additionally, our method is highly expandable in terms of cell types and GHM functions. Recent studies revealed importance of vascular cells and mesenchymal cells in 3D tissue formation^40^,^46^. Combination of our method with other parenchymal cells (hepatocytes, renal epithelial cells and so on) should contribute to the generation of various stem cell-derived large 3D tissues/organs both *in vitro* and *in vivo*. Moreover, GHMs can be modified to have additional functions, such as controlled release of growth factors and small molecules^27^,^47^,^48^. Slow release of angiogenic and/or survival factors should enhance the construct function after TX and integration process to the tissues.

Thus, our method is an important step in the generation of long-lasting cardiac tissue with cell TX and should contribute not only to cardiac cell therapy but also broadly to 3D tissue engineering and regenerative medicine.

## Additional Information

**How to cite this article**: Matsuo, T. *et al.* Efficient long-term survival of cell grafts after myocardial infarction with thick viable cardiac tissue entirely from pluripotent stem cells. *Sci. Rep.* **5**, 16842; doi: 10.1038/srep16842 (2015).

## Supplementary Material

Supplementary Information

## Figures and Tables

**Table 1:** Echocardiogram after 5-cell sheet construct TX to rat myocardial infarction model.

	Group	baseline	pre Tx	1w	2w	4w	8w	12w
LVDd (mm)	Sham	6.4 ± 0.1	7.3 ± 0.2	7.3 ± 0.3	7.8 ± 0.4	8.2 ± 0.5	8.1 ± 0.3	9.2 ± 0.4
	5-Control	6.3 ± 0.2	7.5 ± 0.2	8.2 ± 0.1	8.0 ± 0.1	8.2 ± 0.1	8.9 ± 0.2	9.3 ± 0.3
	5-GHM	6.2 ± 0.1	7.3 ± 0.2	7.6 ± 0.2	7.9 ± 0.2	8.1 ± 0.2	7.4 ± 0.7	8.2 ± 0.4
LVDs (mm)	Sham	3.0 ± 0.1	5.6 ± 0.2	5.8 ± 0.3	6.2 ± 0.3	6.6 ± 0.5	6.6 ± 0.3	7.5 ± 0.4
	5-Control	3.0 ± 0.2	5.8 ± 0.2	6.1 ± 0.2	5.6 ± 0.3	6.2 ± 0.2	6.3 ± 0.2	6.4 ± 0.2
	5-GHM	2.7 ± 0.1	5.5 ± 0.2	5.0 ± ± 0.2^*††^	5.1 ± 0.3	5.1 ± 0.3^*†^	4.1 ± 0.6**	4.5 ± 0.2**
FS (%)	Sham	53.9 ± 1.3	23.1 ± 1.4	20.7 ± 1.0	20.4 ± 1.2	19.1 ± 1.6	18.8 ± 1.3	18.7 ± 1.2
	5-Control	52.3 ± 1.7	23.1 ± 1.6	26.1 ± 1.3*	29.8 ± 2.9*	25.3 ± 1.7	29.5 ± 2.2*	32.1 ± 0.9*
	5-GHM	56.6 ± 1.5	24.9 ± 0.7	34.9 ± 1.5^**††^	36.3 ± 2.1***	37.3 ± 2.7^***††^	45.4 ± 3.9**	44.8 ± 1.7**
Akinetic length (%)	Sham	0.0	24.5 ± 3.3	29.3 ± 2.8	26.0 ± 2.0	28.2 ± 3.6	34.2 ± 5.2	29.2 ± 3.0
	5-Control	0.0	23.1 ± 3.1	14.9 ± 2.8*	13.3 ± 3.0*	16.6 ± 3.2	17.7 ± 1.6	19.1 ± 0.7
	5-GHM	0.0	22.6 ± 1.7	5.5 ± 1.7^***†^	5.4 ± 1.5^***^	0.5 ± 0.4^***††^	0.0^***†^	0.0^**†^

*p < 0.05, **p < 0.01 vs Sham, ^†^p < 0.05, ^††^p < 0.01 vs 5-Control. Kruskal-Wallis test, post-hoc Steel-Dwass multiple comparison test, sham, n = 6; 5-control, n = 11; 5-GHM, n = 10.
