# Supplementary material for: Efficient long-term survival of cell grafts after myocardial infarction with thick viable cardiac tissue entirely from pluripotent stem cells
Source: Sci Rep. 2015 Nov 20;5:16842. doi: 10.1038/srep16842 (PMC4653625; doi:10.1038/srep16842)

Efficient long-term survival of cell grafts after myocardial infarction with thick viable cardiac tissue entirely from pluripotent stem cells

Takehiko Matsuo[1,2,3], Hidetoshi Masumoto[1,2,3], Shuhei Tajima[4], Takeshi Ikuno[1,2,3], Kenji Minakata[3], Tadashi Ikeda[3], Kohei Yamamizu[1,2], Yasuhiko Tabata[4], Ryuzo Sakata[3] & Jun K. Yamashita[1,2*]

[1]Department of Cell Growth and Differentiation, Center for iPS Cell Research and Application (CiRA), Kyoto University, Kyoto, Japan

[2]Laboratory of Stem Cell Differentiation, Stem Cell Research Center, Institute for Frontier Medical Sciences, Kyoto University, Kyoto, Japan

[3]Department of Cardiovascular Surgery, Kyoto University Graduate School of Medicine, Kyoto, Japan

[4]Department of Biomaterials, Institute for Frontier Medical Sciences, Kyoto University, Kyoto, Japan

Legends for Supplementary Figures

Supplementary Fig. S1: Cardiac cell sheet components measured by flow cytometry.

Four days after culture, cardiac sheets were harvested from temperature responsive culture dishes (12-well), and the cellular components were measured by flow cytometry. (a) alpha-MHC-GFP^+ CMs in total cells. (b) $CD31^+$ ECs in the GFP^- non-CM population. $GFP^-/CD31^-$ cells correspond to MCs. Total cell count of the sheet was 3.53±2.1 x 10^5 on average (n=14).

Supplementary Fig. S2: Optimization for dose and diameter of gelatin hydrogel microspheres (GHMs) for the stacking of ESC-derived cardiac cell sheets *in vitro*.

Cardiac cell sheets generated in 24-well UpCell dishes were used. (a-e) Optimization for dose of GHMs. Thicknesses of constructs and tissue viability were evaluated among three groups: control, low dose (0.25 mg GHM/sheet; approximately 0.5 mg/cm^2), and high dose (0.75 mg GHM/sheet). (a-c) HE staining after 7-days culture. (b, c) Black arrowheads indicate GHMs. (d) Thickness of the constructed grafts. Data are shown as mean ± s.e.m. ** $P < 0.01$, * $P < 0.05$ (Kruskal-Wallis test, post-hoc Steel-Dwass multiple comparison test, n = 5). (e) HE- positive cell area in the constructed grafts. Data are shown as mean ± s.e.m. * $P < 0.05$ (Kruskal-Wallis test, post-hoc Steel-Dwass multiple comparison test, n = 4). (f-k) Optimization for size of GHMs. Thicknesses

of constructs and tissue viability were evaluated among four groups: control, φ <20 μm, φ 20-32 μm, and φ 32-53 μm (φ, diameter of GHM). (f-i) HE staining after 7-day culture. Supplementary Fig. S2a and S2f are the same sample as control. Supplementary Fig. S2f and S2h are the same sample with Fig. 2A and 2B, respectively. (j) Thickness of the constructed grafts. Data are shown as mean ± s.e.m. ** $P < 0.01$, * $P < 0.05$ (Kruskal-Wallis test, post-hoc Steel-Dwass multiple comparison test, n = 5). (k) HE-positive cell area in the constructed grafts. Data are shown as mean ± s.e.m. * $P < 0.05$ (Kruskal-Wallis test, post-hoc Steel-Dwass multiple comparison test, control; n = 5, φ <20 μm; n = 4, φ 20-32 μm; n=10, φ 32-53 μm; n = 4). Scale bars, 50 μm.

Supplementary Fig. S3: Extracellular field potential (EFP) measurement

(a-c) Representative images of mesurement procedure. (a) EFP measurement in cell constructs in vitro. The electrodes with 1 mm distance are directly placed onto a cell construct in medium. (b) EFP in cell constructs after TX. After the cell construct was transplanted to a normal rat heart surface, EFP in cell construct was measured by directly placing the electrode on the cell construct. (c) rat electrocardiogram measurement with limb lead electrodes. (d, e) 5-GMH construct TX. Results from the same cell construct before and after TX. (d) Before TX. The construct showed spontaneous and synchronized EFP in vitro (upper panel) with a distinct rhythm from the rat heart beat (lower panel). (e) After TX. The EFP from the construct showed synchronized rhythm with the rat ECG.

Supplementary Fig. S4: Subcutaneous transplantation of GHM sheet constructs *in vivo*.

Subcutaneous transplantation of stacked cell sheets (24-well) into NOD/SCID mouse. HE staining images 7 days after transplantation of 5-GHM construct (right) or 5-control construct (left). Black arrowheads indicate transplanted grafts. Scale bars, 50 μm.

Supplementary Fig. S5: Representative images of perioperative procedure.

(left panel) After exposing a rat heart, a cardiac sheet construct (12-well) was applied to the surface of the heart (white arrowheads indicate the cardiac sheet construct). Then, the construct was spread without folds, to cover the infarcted area and the border area of the LV. (right panel) White dashed circle shows the extent of the cardiac sheet construct. The chest was closed after cardiac cell constructs attached onto the surface of the heart (approximately 20 to 30 minutes later).

Supplementary Fig. S6: GHM constructs promoted host EC recruitment and vascular formation in close proximity of the graft.

Representative images of transplanted cells traced with species-specific (SS)-FISH 1 week after GHM construct transplantation. Triple staining for cTnT (red), vWF (green), mouse nuclei

(yellow), and DAPI (blue). (a) Gross appearance of infarcted area. Left panel: control. Right panel: GHM construct. White dashed lines enclose the engrafted constructs. Scale bar, 1 mm. (b) High-magnification images of the border area between the host and graft. Left panel: control. Right panel: GHM construct. White arrowheads indicate host ECs (non-yellow nuclei). Vascular structures outside the graft are mainly formed with host ECs. Scale bar, 250 μm. (c) High-magnification image of the yellow box in (b). Graft-derived CMs (red/yellow) and ECs (green/yellow) are observed. Scale bar, 50 μm.

Supplementary Fig. S7: cTnT-negative cells in the grafts are SMA-positive cells.

Immunofluorescent staining of heart sections 7 days after TX. (a) Double staining for cTnT and vWF. (b) Double staining for αSMA and vWF (from a serial section in (a)). White dashed lines indicate boundary between host and graft. White dotted lines indicate boundary between cTnT-negative and cTnT-positive areas in the graft. Note that almost all the cTnT-negative area is positive for SMA. Nuclei were stained with Hoechst 33342 just before TX. Scale bars, 100 μm.

Supplementary Fig. S8: Generation of hiPS-derived GHM constructs *in vitro*.

5-control construct (left) and 5-GHM construct (right) generated in 12-well UpCell dishes. HE staining after 7 days culture. Scar bars, 100 µm.

SUPPLEMENTARY FIGURE 1

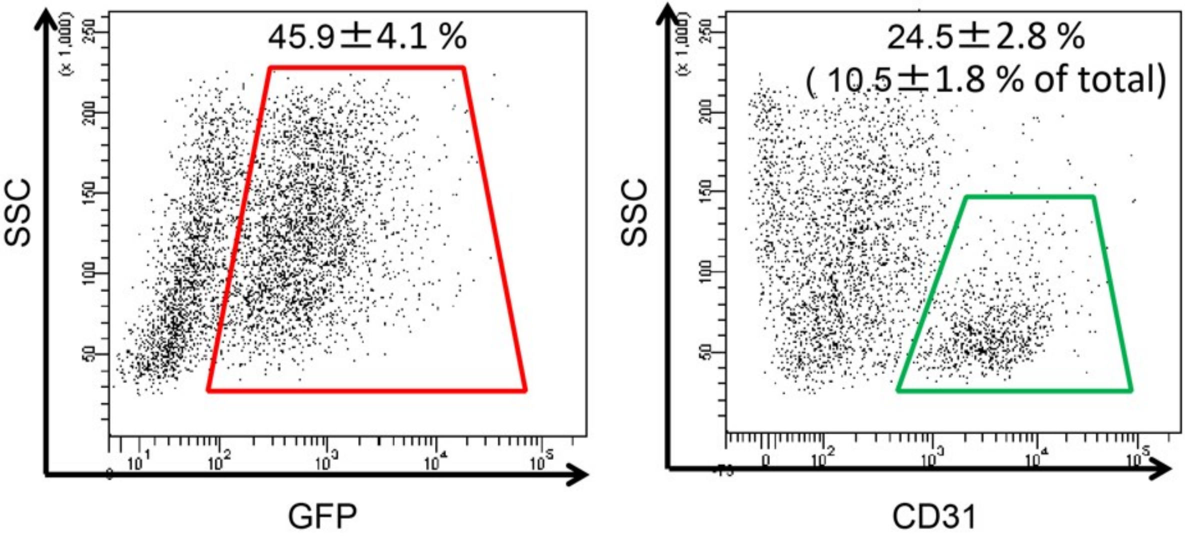

Cell count: 3.53±2.1 x10^5 /sheet

SUPPLEMENTARY FIGURE 2

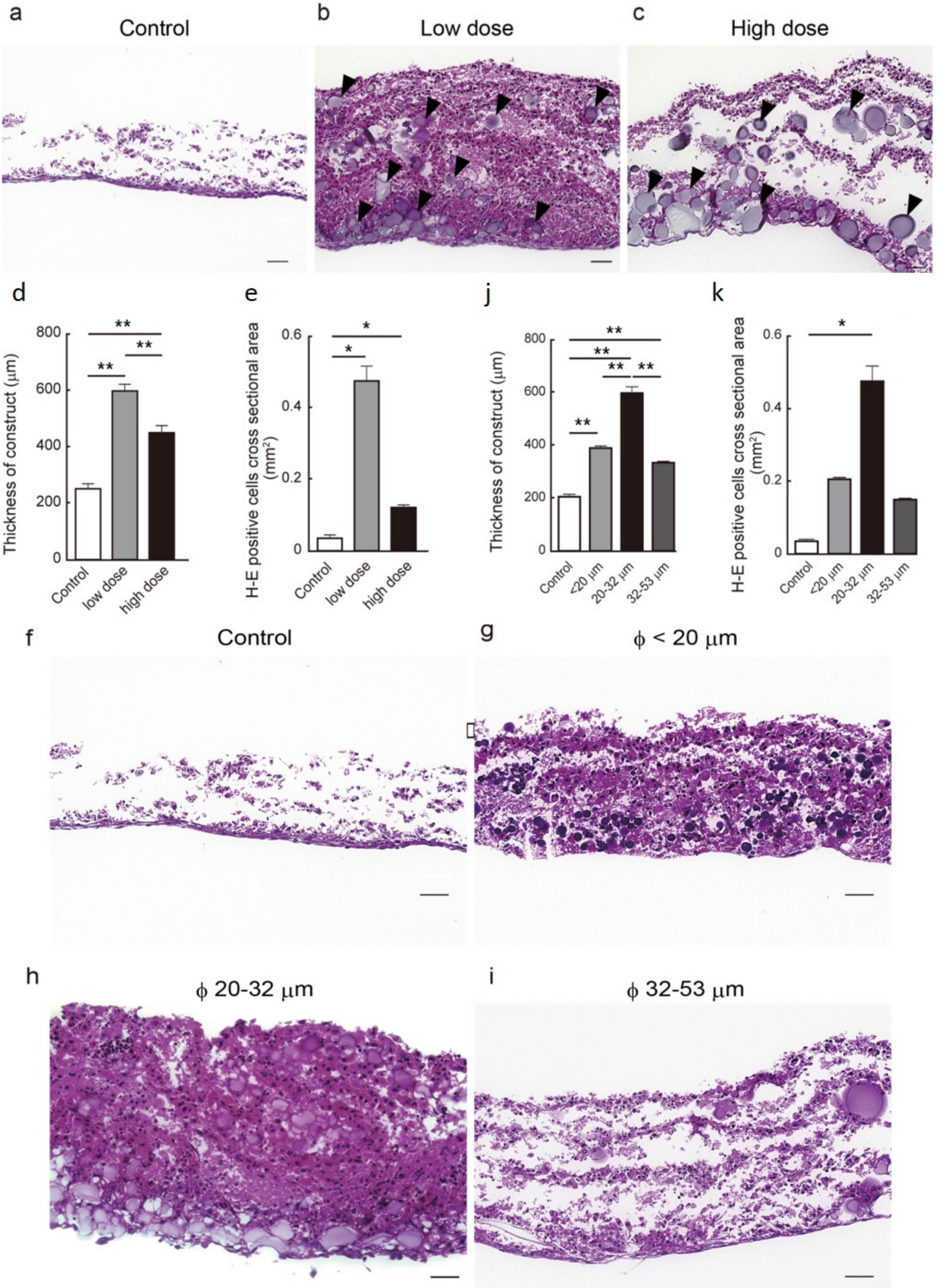

SUPPLEMENTAL FIGURE 3

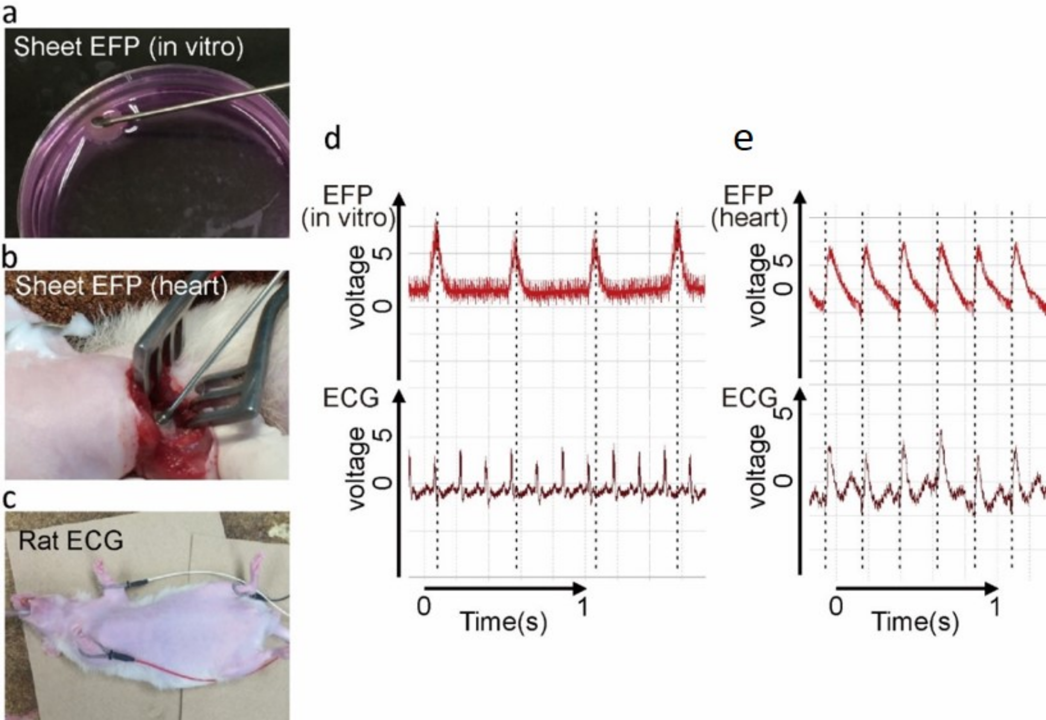

SUPPLEMENTARY FIGURE 4

5-Control 5-GHM

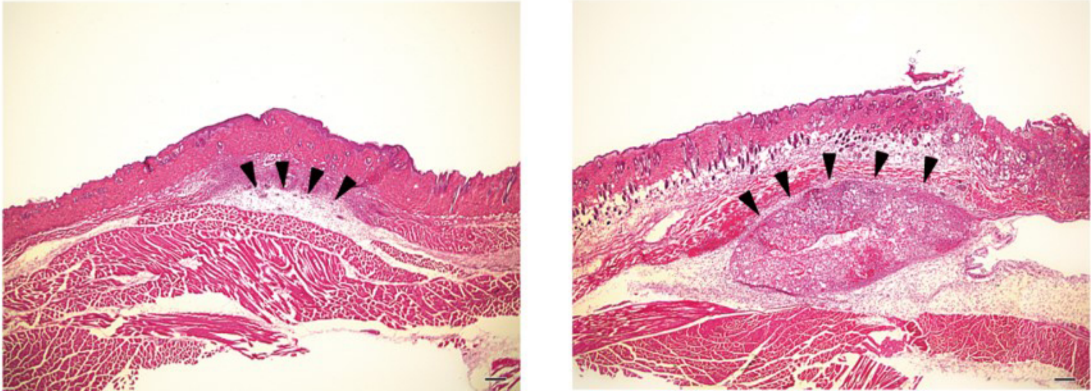

SUPPLEMENTARY FIGURE 5

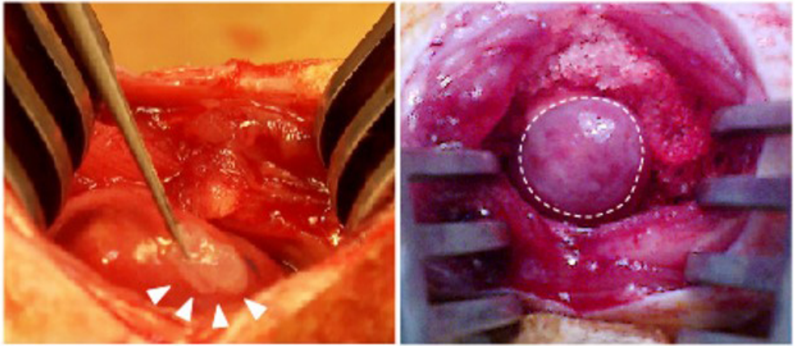

SUPPLEMENTAL FIGURE 6

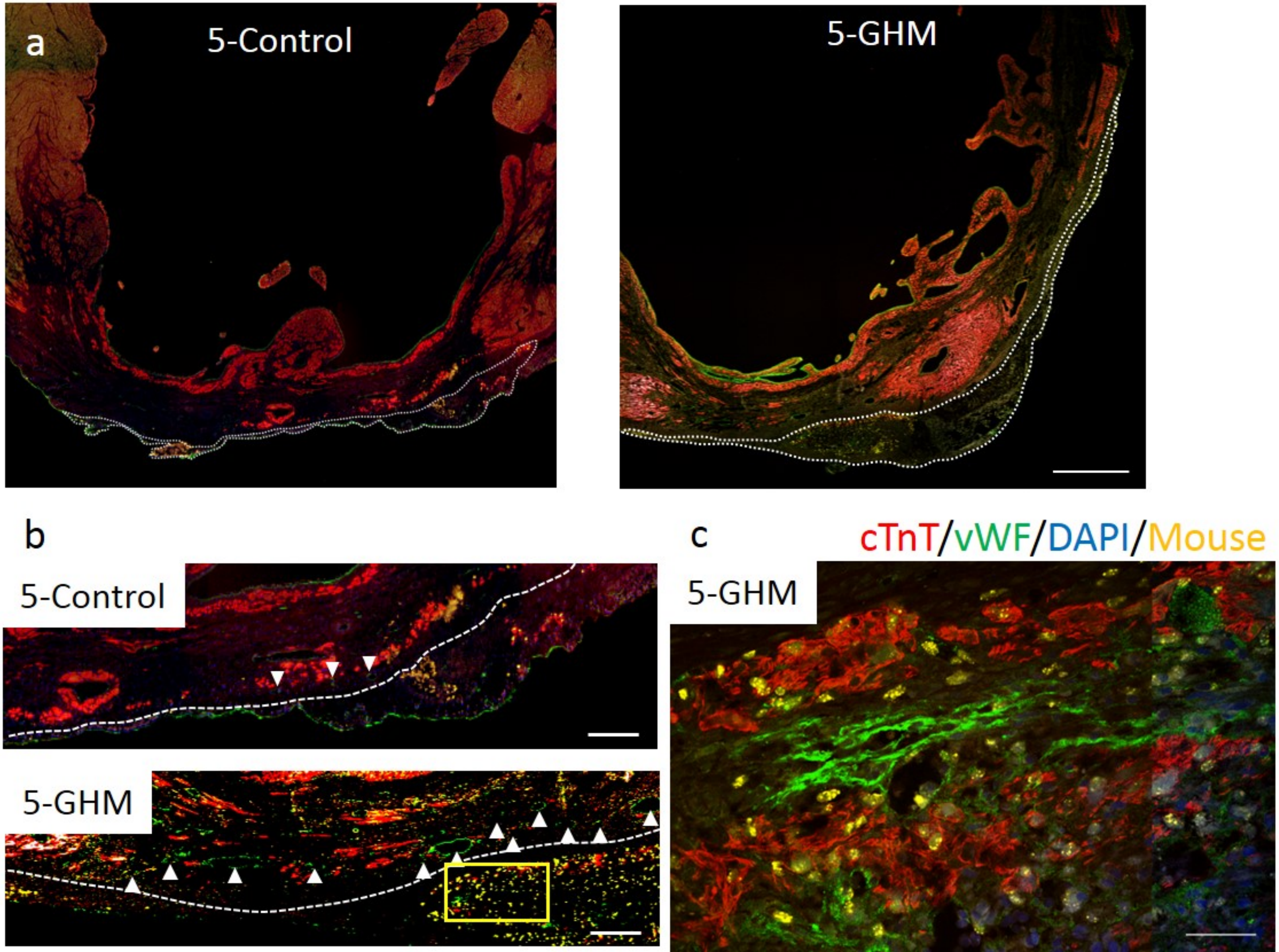

SUPPLEMENTARY FIGURE 7

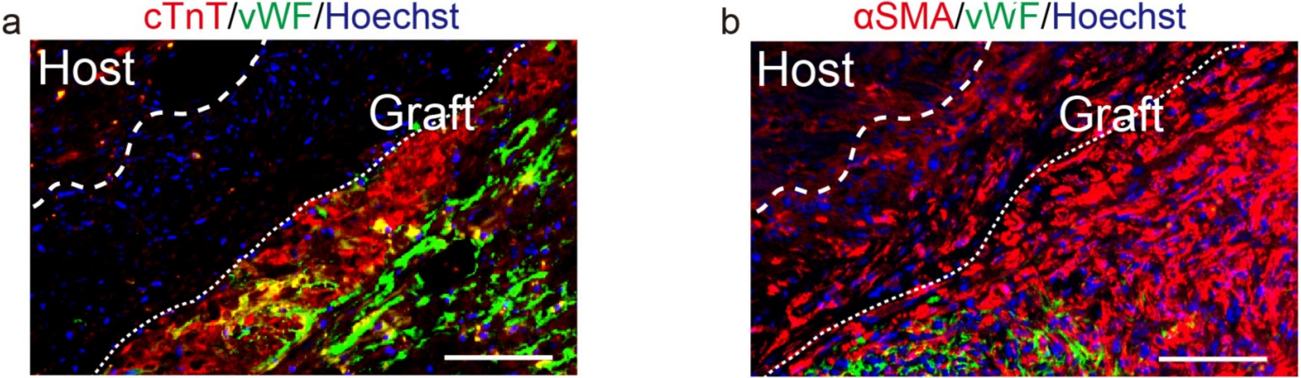

SUPPLEMENTARY FIGURE 8

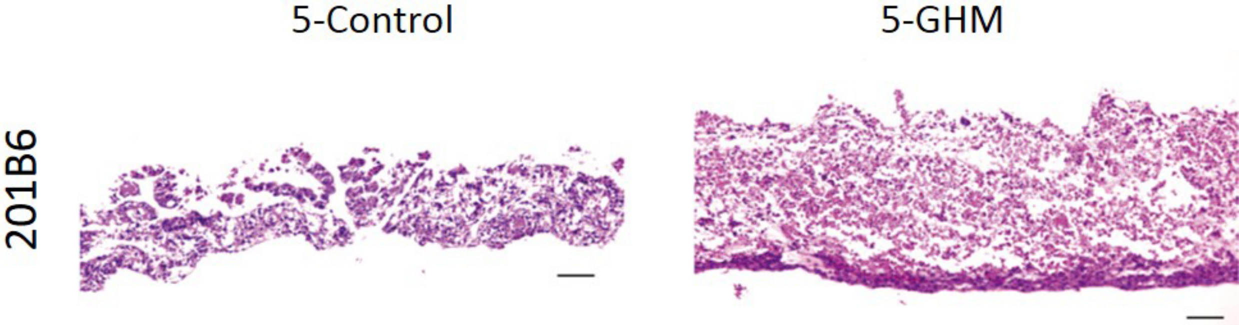

Supplement: Supplementary Information [file srep16842-s1.pdf]
